# Disney Rash in Las Vegas: Benign Lower Extremity Purpura Following Prolonged Walking

**DOI:** 10.7759/cureus.67298

**Published:** 2024-08-20

**Authors:** Juliette Camejo, Margaret Savage, Dana Hernandez, Vijay Rajput

**Affiliations:** 1 Internal Medicine, Nova Southeastern University Dr. Kiran C. Patel College of Allopathic Medicine, Davie, USA; 2 Medical Education, Nova Southeastern University Dr. Kiran C. Patel College of Allopathic Medicine, Davie, USA

**Keywords:** exercise-induced purpura, disney rash, golfer's vasculitis, small vessel vasculitis, palpable purpura, lower extremity purpura, exercise induced vasculitis, vasculitis

## Abstract

This case follows an adult middle-aged female patient who developed a purpuric rash, soreness, and swelling on her legs after walking for several days in Las Vegas. With no prior petechial rash history or presence of systemic symptoms, exercise-induced purpura (EIP) was suspected due to her protracted walking in warm weather. She recovered fully with supportive treatment. EIP, also known as exercise-induced vasculitis (EIV), is often poorly recognized, misdiagnosed, and inappropriately treated. There is a need for increased awareness and clinical diagnosis of EIV based on thorough history and physical examination.

## Introduction

Purpura in adults typically indicates an underlying blood clotting disorder or multi-system autoimmune disease requiring urgent attention. This skin manifestation is the most common physical sign of cutaneous vasculitis, the pathological process that results from inflammation of skin blood vessels leading to alterations in blood flow, ischemia, and damage to the tissue [1].

Exercise-induced purpura (EIP), a form of benign small-vessel cutaneous vasculitis, mostly occurs on healthy-appearing individuals after prolonged walking in hot climates and is often referred to as exercise-induced vasculitis (EIV) [2, 3]. Extended periods of walking in warm weather commonly take place in settings ranging from marathon races to amusement and national parks. Because it occurs in a vast range of locations, EIP is commonly known by a variety of names, including Disney Rash, golfer’s rash/vasculitis, hikers’ dermatitis/purpura, jogger’s petechiae, sport-induced vasculitis, and marathon runner’s vasculitis. Although a result of warm weather and prolonged walking, sun exposure is not an influencing factor [4]. Additionally, areas of compression, such as under the sock, are spared from visible signs of purpura [5]. EIV has a self-limiting course and systemic symptoms are usually absent [6]. EIV is often misdiagnosed as other common conditions of the lower legs; therefore, this report aims to contribute information to the presentation, diagnosis, and treatment of EIV described in this case.

This article was previously submitted as an abstract to the 2023 ACP Florida Chapter Annual Meeting in October of 2023 and as a poster to the LMSA SE Regional Conference 2024: Estetoscopio Latino, Corazón Que Escucha in February 24, 2024.

## Case presentation

A 55-year-old post-menopausal Asian woman from Europe with a sedentary lifestyle traveled to Las Vegas for a vacation. She had a history of hypertension and type 2 diabetes mellitus controlled with enalapril 20mg once daily and metformin 500mg twice daily, respectively, for the last five years. She noticed a purpuric rash with mild soreness and edema on her lower extremities, extending from above the ankles to the mid-lower legs at the end of her four-day trip. She was wearing knee-length shorts. There was no prior history of petechial rash or easy bruisability. She did not have a prior history of any rheumatological and liver diseases. She reported consumption of a few glasses of wine daily and denied ingestion or handling of raw fish, oysters, or meat. She also denied taking any other medications or supplements. She walked 20,000-30,000 steps every day, for a total of four days, as registered on a wearable lifestyle device. She wore sneakers and ankle-socks throughout her trip. Additionally, she denied the following symptoms: fever, sore throat, cough, wheezing, abdominal pain, diarrhea, hemoptysis, and changes in urine color. 

**Figure 1:**
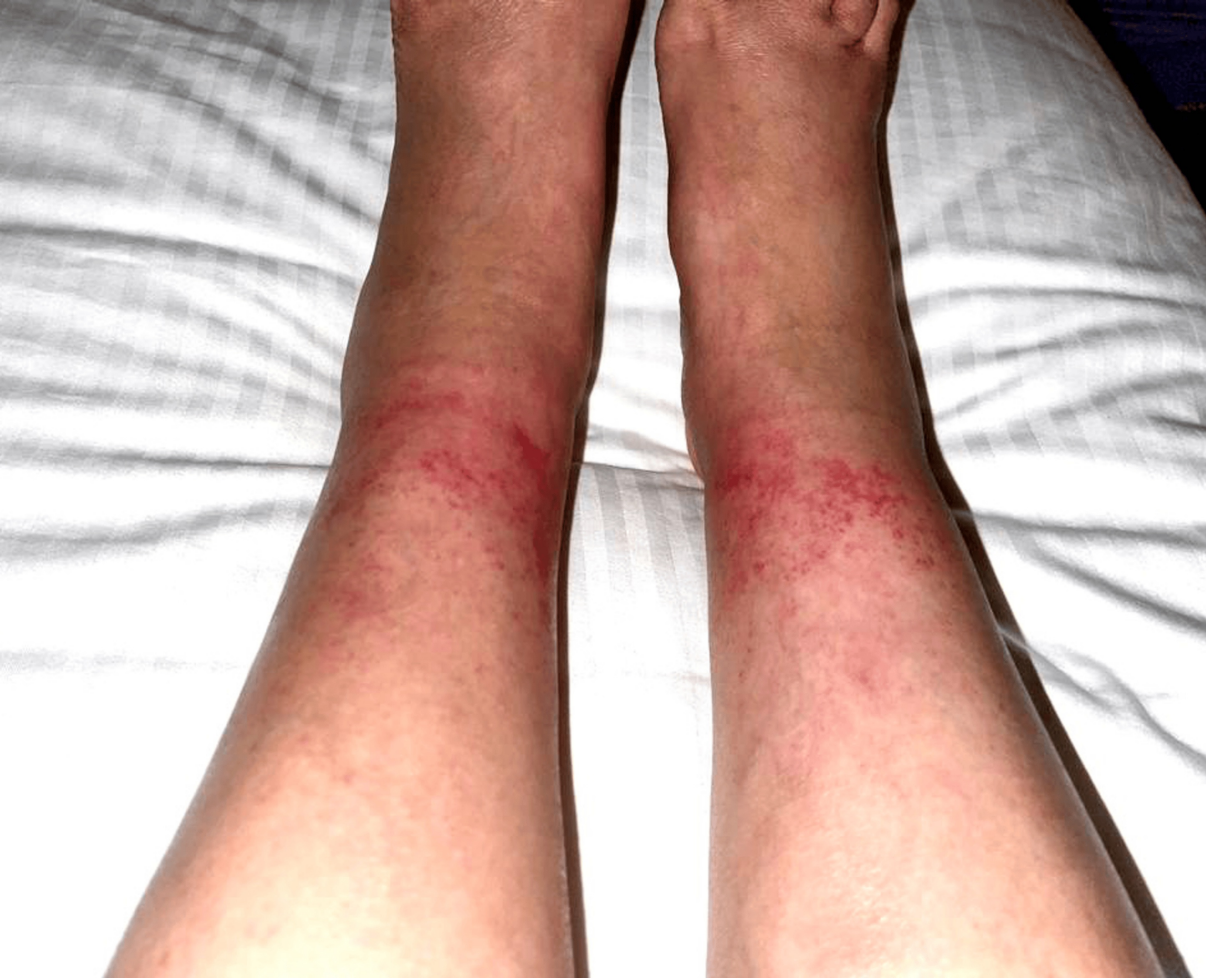
Initial presentation: erythematous purpuric rash on bilateral lower extremities, area covered by ankle socks was spared

**Figure 2:**
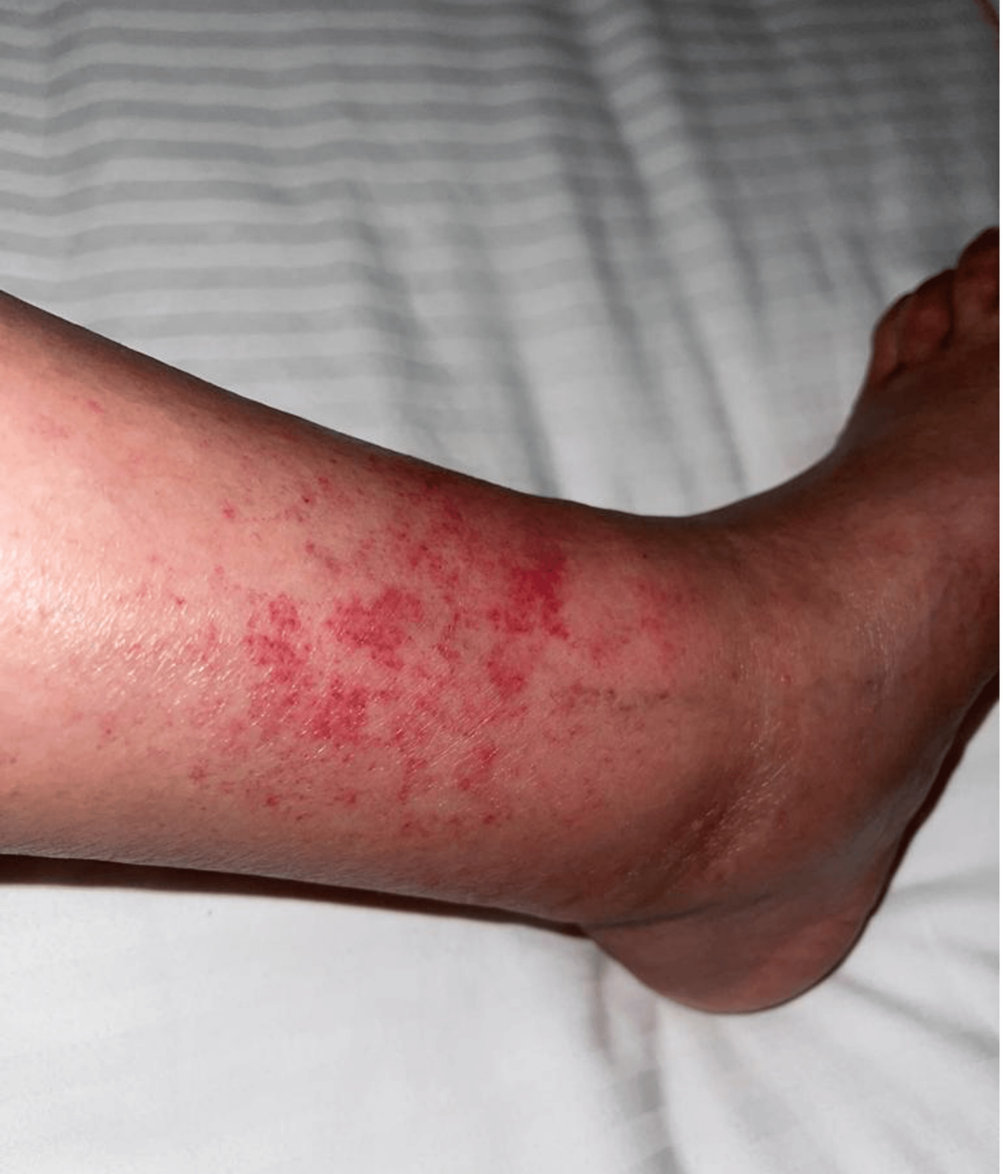
Initial presentation

**Figure 3:**
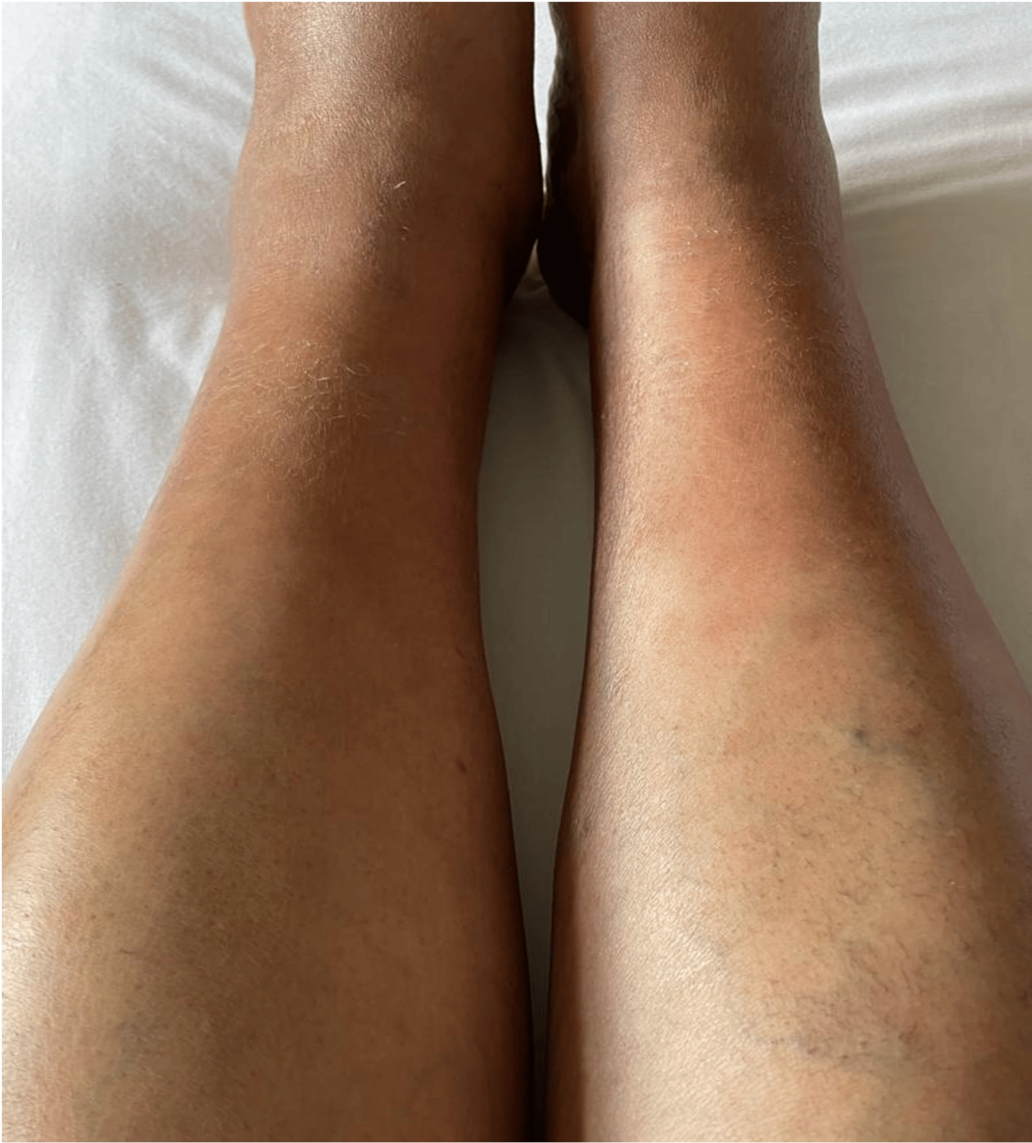
Three weeks after the initial presentation

**Figure 4:**
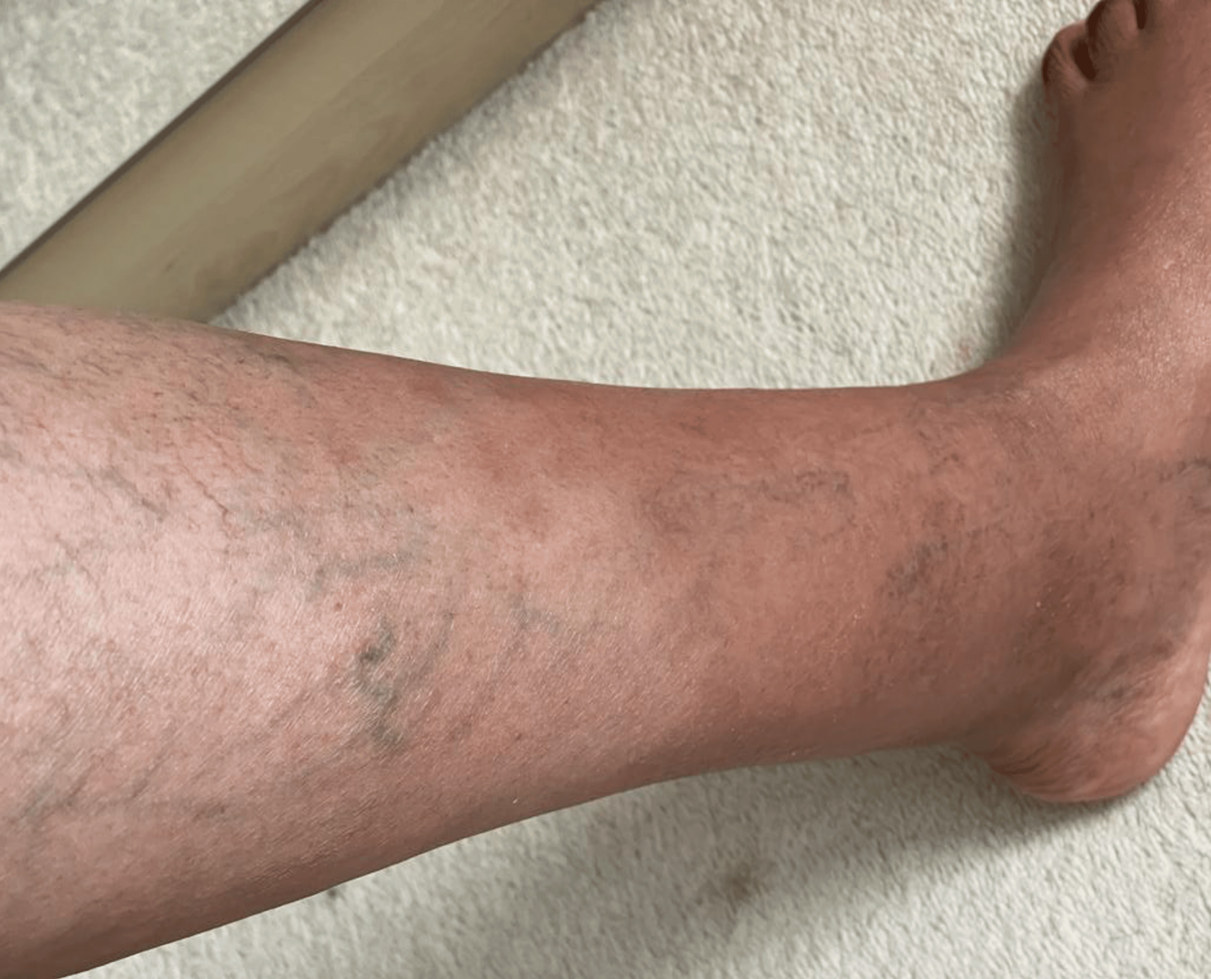
Three weeks after the initial presentation, superficial varicose veins seen (previous finding)

She was afebrile. Her BMI was 27. Her physical exam was unremarkable apart from the lower-extremity palpable, petechial, and purpuric rash that presented above the ankle and extended proximally for 5 cm (Figures 1, 2). The area covered by ankle socks was spared of any findings. Based on her history of prolonged walking and absence of systemic symptoms, a clinical diagnosis of EIP was made. Laboratory testing was not performed. She recovered (Figures 3, 4) with a supportive care regimen of elevation, cold compression, and heat avoidance for three weeks.

## Discussion

Exercise-induced purpura was initially documented in the 2000s dermatology literature, described as a pruritic, burning lower extremity rash following prolonged walking in warm climates. Since then, EIP has been labeled in multiple contexts of activities or geographical locations as mentioned previously. The incidence of EIP/EIV in the current literature remains unknown.

EIV has been shown to predominantly affect women in their 50s with no significant medical history. Although typically painless, symptoms may include pruritus, pain, and a burning sensation [2]. While it most commonly affects the lower extremities, it has been documented to impact the forehead and periorbital regions, as well as the back and trunk [3, 7-8]. EIP is an under-reported disorder that resolves spontaneously within 10-14 days and is often easily misdiagnosed. This indicates a need to raise awareness among primary care physicians and allied health professionals.

Clinical pathophysiology

This clinical presentation is common and generally uniform following physical triggers, including heat exposure and prolonged periods of exercise, including, but not limited to, hiking, golfing, jogging, and walking. Despite EIV’s prevalence amongst middle-aged females, its underlying pathophysiology is not fully understood. The prevailing mechanism is thought to be driven by the intersection of various components, incorporating thermoregulation impairment, arterial remodeling, and immune system activation [2, 4, 9]. In a 2016 systematic review, it was proposed that dysregulation of heat in lower extremity muscles in warm weather leads to continuous vasodilation and ultimately erythrocyte extravasation [4]. Additionally, the presence of lipedema and orthostatic edema in the legs may further contribute to the accumulation of heat and the onset of EIV [2].

The role of chronic venous disease (CVD) in EIV remains controversial. While Ramelet discounts CVD as a potential risk factor [2], it has been described as having a significant role in the development of EIV [9]. The study, conducted in 2022, showed a significant association of CVD in EIV compared to the control group of hikers [9]. Using the Clinical Etiology Anatomy Pathophysiology (CEAP) classification system for describing patients with chronic venous disorders (Table 1), this same group of patients also experienced significantly more C2/3 venous and greater saphenous venous insufficiencies present in the EIV cohort [9]. C2 is used to describe varicose veins, while C3 is used to signal edema [10]. It is currently thought that CVD may exacerbate the exercise-induced vasodilatory effect, causing an overflow of blood in the venous system. This induces extravasation of red blood cells and the resulting purpura [4, 11].

The increased volume of blood may induce additional physiological changes secondary to endothelial stress, including remodeling of small arteries and the release of inflammatory markers [9, 12]. It has been suggested that the exercise-induced vasodilatory effect can initiate the atherosclerotic process, thereby contributing to plaque formation and arterial remodeling that is driven by an inflammatory process [9, 12]. This can be visualized by the accumulation of reactive oxygen species, metalloproteinases, and cytokines, as well as immune complexes secondary to complement activation [9,11,12].

**Table 1:** CEAP Classification System and Reporting Standard Revision 2000 CEAP: Clinical manifestations, etiology, anatomic distribution, pathophysiology Source: Lurie et al. [10].

CEAP Classification System and Reporting Standard Revision 2000	
C0	No visible or palpable signs of venous disease
C1	Telangiectasias or reticular veins
C2	Varicose veins
C2r	Recurrent varicose veins
C3	Edema
C4	Changes in skin and subcutaneous tissue secondary to chronic venous disease
C4a	Pigmentation or eczema
C4b	Lipodermatosclerosis or atrophie blanche
C4c	Corona phlebectatica
C5	Healed
C6	Active venous ulcer
C6r	Recurrent active venous ulcer

Clinical diagnosis

Exercise-induced vasculitis is a condition that is predominantly diagnosed clinically based on symptomatology and attributed to a history of prolonged walking. Differential diagnoses can be broad, including stasis dermatitis, cellulitis, pigmented purpuric dermatoses, and other forms of benign vasculitis, such as Henoch-Schoenlein purpura. What primarily distinguishes EIV from these etiologies of petechiae is the frequent relapse in triggering conditions like prolonged walking in hot climates. Further features of the physical exam that support an EIV diagnosis include a sparing of the purpura within the sock-compressed areas [5]. Additionally, while EIV is usually present in a circumferential manner in the lower legs, stasis dermatitis is frequently restricted to the medial malleolus [11].

The patient did not have a history of easy bruisability, petechial rashes, or any significant family history of bleeding disorder, which makes the diagnosis of a genetic platelet or bleeding disorder unlikely. The absence of clinical sepsis syndrome and lack of raw fish consumption excluded the possibility of a vibrio vulnificus infection. The absence of systemic symptoms, such as fever, ruled out the possibility of an allergic reaction, as well as potential cellulitis. Additionally, the risk factors for developing erysipelas/cellulitis caused by *Streptococcus pyogenes*, including handling of raw fish or meat, were all denied by the patient [13]. The remainder of the physical exam remained unremarkable. Due to the clinician having previous experience of diagnosing patients with EIV, no laboratory testing was performed and diagnosis was made based on history and clinical findings.

There are a variety of additional diagnostic laboratory tests that can be used, including blood counts, complement studies, and histological analyses. Complete blood counts, as well as C3/C4 complement level and antinuclear antibodies, are typically within normal limits [4, 6]. Histological analysis can reveal C3 or immunoglobulin M (IgM) deposits within a vessel wall as small vessel leukocytoclastic or urticarial vasculitis [4]. The role of Duplex ultrasound has been suggested as a potential tool for evaluating the underlying venous thrombosis in patients with EIV [2].

Treatment

The resolution of EIV typically occurs spontaneously, with full remission within seven to 10 days. To prevent relapses, preventative measures can be implemented, including the use of compression stockings for better venous and lymphatic drainage [2]. The role of medications, such as topical steroids, nonsteroidal anti-inflammatory drugs (NSAIDs), and antihistamine agents, remains uncertain. More specifically, while prior case reports have exhibited the use of steroids for the treatment of EIV, the current scientific literature does not contain a study that has compared the efficacy of treatment plans utilizing steroids versus no steroids in preventing EIP relapse [2, 5, 6]. As a result, further investigation is needed to conclusively make a recommendation for EIV treatment regimens. 

## Conclusions

EIP continues to be an under-reported condition that predominantly affects healthy women in their 50s. EIP has been described as Disney rash, golfer's vasculitis, and by many other names. This benign, yet often misdiagnosed, condition is self-limited and is associated with small vessel leukocytoclastic vasculitis, although more research is needed to strengthen this association.

The preventative measures for EIP include the use of compression stockings and lymphatic drainage, although the efficacy of medications such as topical steroids, NSAIDs, and antihistamines remains uncertain. Misdiagnosis of EIP as conditions like stasis dermatitis or cellulitis underscores the importance of recognizing bilateral lower extremity purpura in patients following prolonged exercise in warm weather as potential signs of EIP/benign EIV. The awareness of this clinical condition is crucial to avoid unnecessary diagnostic tests, inappropriate treatments, or hospitalizations.
